# Glucagon- and insulin-immunopositive endocrine cells in porcine extrahepatic bile ducts and gallbladder

**DOI:** 10.3389/fvets.2023.1240143

**Published:** 2023-11-29

**Authors:** Ivaylo Stefanov Stefanov, Stefan Ivaylov Stefanov, Maya Vladova Gulubova

**Affiliations:** ^1^Department of Anantomy, Medical Faculty, Trakia University, Stara Zagora, Bulgaria; ^2^Department of Anatomy, Histology and Embryology, Pathology, Prof. Dr. Asen Zlatarov University, Burgas, Bulgaria; ^3^Medical Faculty, Sixth Year Student, Trakia University, Stara Zagora, Bulgaria; ^4^Department of General and Clinical Pathology, Medical Faculty, Trakia University, Stara Zagora, Bulgaria

**Keywords:** glucagon, insulin, endocrine cells, bile ducts, gallbladder, pig

## Abstract

**Introduction:**

Pancreatic β-cells and α-cells have been found in the murine extrahepatic biliary ducts but not in the gallbladder. However, there has been no information reported in the specialized literature about the presence of glucagon- and insulin-expressing endocrine cells in porcine bile ducts and gallbladder.

**Aim:**

We aimed to perform an immunohistochemical study to identify glucagon- and insulin-positive cells and their distribution in the porcine extrahepatic biliary ducts and gallbladder.

**Method:**

The immunohistochemical method was used to detect the presence and distribution of glucagon- and insulin-positive endocrine cells in the common hepatic duct (*ductus hepaticus communis*), common bile duct (*ductus choledochus*), cystic duct (*ductus cysticus*), and gallbladder (*vesica fellea*) of male pigs. Chromogranin A was used as a typical marker for endocrine cells.

**Results:**

The density of chromogranin A-, glucagon- and insulin-positive cells per field was the largest in the common bile duct, followed by the common hepatic duct, cystic duct, and gallbladder. The three types of endocrine cells showed specific localization in the superficial and deep glands of the studied organs.

**Conclusion and clinical importance:**

The distribution of glucagon- and insulin-immunopositive endocrine cells in the porcine extrahepatic biliary tract was established for the first time as a new source of these hormones. The presence of α- and β-cells in the epithelium of extrahepatic bile ducts can be applied in treatment of diabetes, taking into account the possibility to reprogram the biliary epithelium to mentioned pancreatic endocrine cell types.

## Introduction

Due to the similarity in body weight, anatomy, and physiology between pigs and humans, the pig has become a model for morphological, physiological, biochemical, and genetic investigations (1).

The anatomical and physiological similarities of organs such as the liver, pancreas, kidney, and heart have also made the pig a major species of interest as an organ donor for xenotransplantation procedures (2, 3).

Despite minor morphological differences, swine are the ideal species for liver xenotransplantation. Modern medical advances have enabled xenotransplantation of the liver from genetically modified pig donors. New medical techniques could make xenotransplantation an important method to solve the problem of providing livers from human donors (3–7).

It is well known that extrahepatic bile ducts are represented by the common hepatic duct (*ductus hepaticus communis*, DHC), the common bile duct (*ductus choledochus*, DCH), and the cystic duct (*ductus cysticus*, DC). The gallbladder (*Vesica biliaris*, VB) collects bile and releases it through the cystic duct into the common bile duct (8, 9).

Considering the common origin of the pancreas and gallbladder together with extrahepatic bile ducts (10–12), it is important to know if the porcine biliary tract has endocrine cells producing insulin and glucagon which can contribute to the pathophysiology and treatment of diabetes. Some authors like Dutton et al. (13) described a population of pancreatic-like endocrine cells which are localized in murine extrahepatic bile ducts. The insulin-positive cells were described as situated between cholangiocytes of bile ducts, but not between cholangiocytes of the gallbladder. They were defined as β-cells producing insulin (14). The common bile duct’s ability to generate pancreatic cells is explained mainly by the common embryologic development of bile and pancreatic ducts (8, 12, 14–16). According to Shiojiri (17), the common bile duct developed from the caudal hepatic foregut endoderm near the pancreatic primordium (18–20). Other authors such as Terada et al. (21), found out that pancreatic amylase was expressed in both hepatocytes and cholangiocytes of the primitive hilar bile ducts (21). The endocrine pancreas in the sea lamprey was established to have arisen through the transdifferentiation of the common bile duct (22, 23).

In mice, it was established that the origin of endocrine cells are from the biliary duct epithelium but not from the pancreas (8, 12, 16). It was concluded that, according to their origin, biliary β-cells differ from pancreatic ones.

A new approach in islet transplantation therapies for type 1 diabetes based on the production of β-like cells *in vitro* has been developed. This may involve the use of islet progenitor cells, adult and embryonic stem cells, and mature β-cells (24–27). Another method is to produce β-cells from cells belonging to tissues with similar origin to the pancreas. Several authors have managed to convert hepatocytes to β-like cells (26, 28–31). Therefore, β-cells of the DHC, DCH, DC, and VB could be used for the treatment of diabetes, but more studies should be performed in this direction.

We aimed to describe for the first time the localization and density of glucagon- (Glu^+^Cs) and insulin (Ins^+^Cs)-immunopositive endocrine cells in the wall of porcine DHC, DCH, DC, and VB for better understanding of the pathophysiology and treatment of diabetes.

## Methods

### Animals

The present study used six clinically healthy male 6 months old pigs (Bulgarian White × Landrace cross) (92–100 kg) supplied from a single commercial farm, subjected to a standard age-appropriate diet and slaughtered in a regulated abattoir approved by the Bulgarian Food Safety Agency, and was funded by the Scientific Project number 13/2017, Medical Faculty, Trakia University, Stara Zagora, Bulgaria. Six porcine livers, together with the gallbladder, cystic duct, common hepatic duct, common bile duct, and duodenum, were collected from pigs intended for meat consumption at the slaughterhouse. Tissue samples were obtained from the common hepatic duct (DHC), the initial part of cystic duct (DC) near the gallbladder’s neck, the initial part of the common bile duct (DCHO) near the junction with the cystic duct, the intramural part of common bile duct (DCHI), and the gallbladder’s neck immediately after slaughtering and fixed in a 10% aqueous solution of formalin.

Routine histological techniques were used to process the material and to obtain serial paraffin sections that were stained with hematoxylin and eosin to exclude the presence of pathological findings. Another part of the sections was processed immunohistochemically for detection of glucagon and insulin expression.

### Immunohistochemical method for visualization of glucagon- and insulin-positive endocrine cells compared with chromogranin A-positive endocrine cells

In this work, the ABC (avidin-biotin peroxidase complex) technique was performed. Serial tissue sections with 5 μm thickness were washed in 0.1 M PBS and placed in 1.2% hydrogen peroxide in methanol for 30 min. Antigen retrieval in buffer (pH 9.0) was done for 20 min. Between steps, sections were washed with an EnVision Flex Wash Buffer, then incubated in a humidified chamber overnight at 4°C with primary antibodies: glucagon mouse monoclonal antibody (1:50 dilution in PBS, (C-11) SC-514592, Santa Cruz Biotechnology, Dallas, TX, United States), insulin mouse monoclonal antibody (1:50 dilution, (2D 11–45), SC-8033, Santa Cruz), and chromogranin A rabbit antibody (PA 0430) (Leica Microsystems Inc.), which were ready to use. The immune reaction was visualized with diaminobenzidine. Three serial sections on a slide were stained consequently with glucagon-, insulin-, and chromogranin A antibodies. Three slides per animal were used.

PBS is used instead of primary antibody as a negative control.

### Statistical analysis

The number of endocrine cells was estimated on three microscopic fields X100 from three sections of the DHC and extra- and intramural parts of the DCH, DC, and gallbladder’s neck for each antibody and per each animal. The data for endocrine cell density (number of endocrine cells per field and per cross section of a gland) were processed by Graph Pad Prism 6 for Windows (Graph Pad Software, Inc., United States) via one-way ANOVA followed by the Tukey–Kramer post-hoc test. *p*-values of less than 0.05 were considered statistically significant. The data are presented as mean ± SD.

## Results

### Immunoexpression of chromogranin A

**Figure 1:**
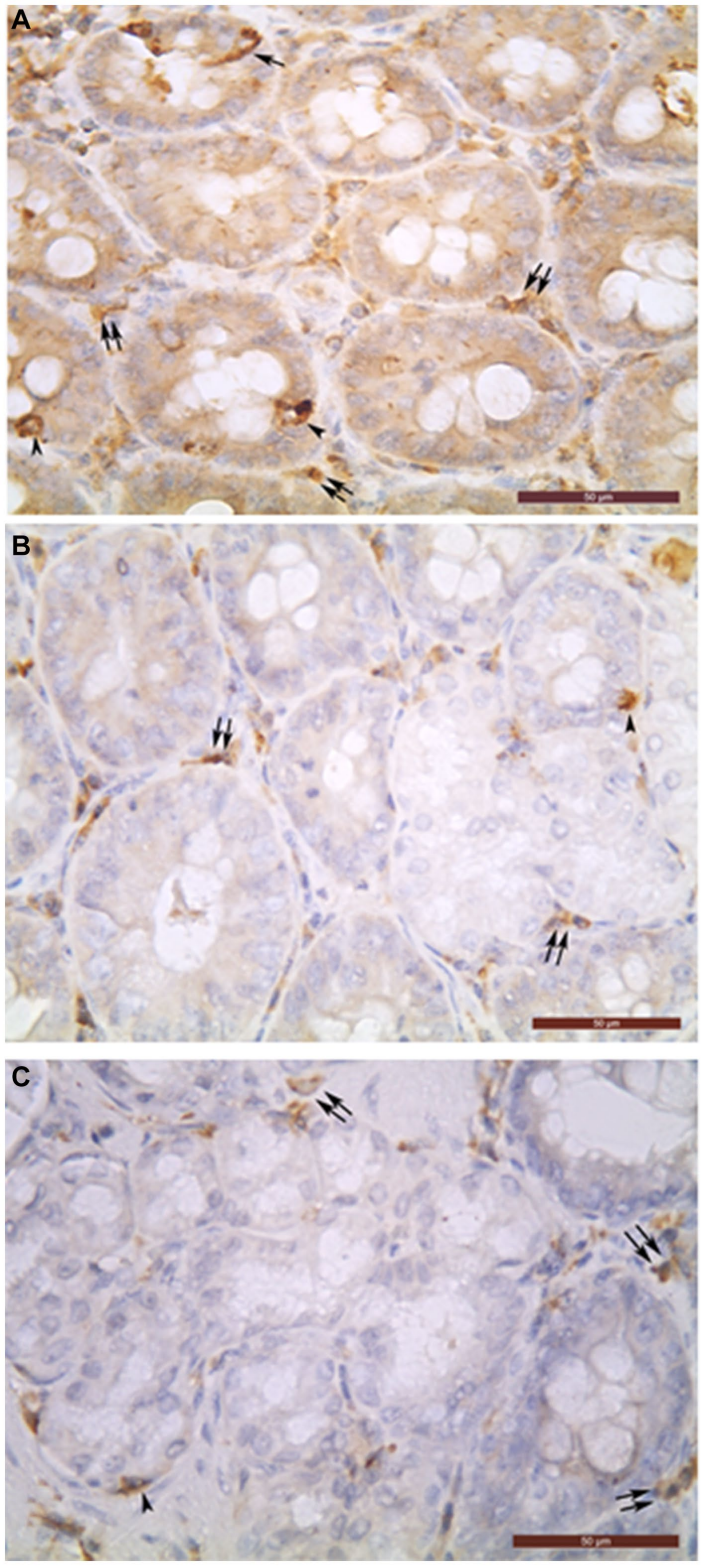
Chromogranin A-positive cells **(A)**, glucagon-positive cells **(B)** and insulin-positive glandular cells **(C)** in the intramural part of common bile duct (**A–C**). Open (arrowheads) and closed (arrows) types of endocrine cells can be seen in the biliary glands. In the interglandular connective tissue, many chromogranin A- **(A)**, glucagon- **(B)**, and insulin- **(C)** immunoreactive cells were detected as well (double arrows). Bar = 50 μm.

Immunoexpression of chromogranin A (ChA) was used as a marker for endocrine cells to detect all endocrine cells in the studied organs. In this manner, we identified the largest number of endocrine cells (ChrA^+^Cs) in the glands of the gallbladder and extrahepatic bile ducts (Figure 1 and Table 1). The density of ChrA^+^Cs per field was largest in the DCHI, followed by DCHO, DHC, DC, and VB (Table 1).

**Table 1:** Number of chromogranin A-, insulin-, and glucagon-positive cells (ChrA^+^C, Ins^+^C, and Glu^+^C, respectively) per microscopic field or per cross section (average number of sections from all animals) of a gland in the gall bladder (VF), *ductus cysticus* (DC), *ductus hepaticus communis* (DHC), initial segment of *ductus choledochus* (DCHO), and intraduodenal part *of ductus choledochus* (DCHI) represented as mean ± SD (standard deviation).

Endocrine cell types	DHC mean ± SD	DCHO mean ± SD	DCHI mean ± SD	DC mean ± SD	VF collum mean ± SD
ChrA^+^C/F	5.00 ± 0.76 A4, B4, C2, D4, b4, c4	7.50 ± 0.51 E4, F4, G4	59.94 ± 3.55 H4, I4	3.05 ± 0.63 J4	1.00 ± 0.00
Ins^+^C/F	2.11 ± 1.27 d0 A4, B4, C0, D4	3.61 ± 0.50 d4 E4, F2, G4	4.83 ± 0.85d0 H4, I4	2.72 ± 0.46 d0	1.00 ± 0.00 d0
Glu^+^C/F	1.94 ± 0.93 A0, B4, C0, D4	2.11 ± 0.83 E4, F0, G4	5.22 ± 0.80 H4, I4	1.83 ± 0.38	1.00 ± 0.00
ChrA^+^C/GL-SGls-DGls	A2, B4, C0, D0 1.33 ± 0.48 a4 2.33 ± 0.48 A4,B0,C4	E4, F4, G4 2.11 ± 0.75 a4 3.22 ± 0.73 E0, F4, G4	H4, I4 7.27 ± 0.75 a4 2.66 ± 0.48 H4, I4	J0 1.16 ± 0.38 a0 1.11 ± 0.32 J0	1.00 ± 0.00
Ins^+^C/GL-SGls-DGls	A0, B4, C0, DO 1.50 ± 0.78	E4, F2, G2 1.94 ± 0.80	H4, I4 3.44 ± 0.51 a4 1.33 ± 0.48	1.11 ± 0.32	1.00 ± 0.00
Glu^+^C/GL-SGls-DGls	A3, B4, C0, D0 1.11 ± 0.32	1.94 ± 0.80	3.44 ± 0.51 a4 1.61 0.50	1.11 ± 0.32	1.00 ± 0.00
ChrA^+^Cot/F	1.4 ± 0.51 A2, B4, C0	2.50 ± 0.51 E4, F1	39.44 ± 1.19 H4, h4, i4	1.50 ± 0.51	–
Ins^+^Cot/F	–	–	2.27 ± 0.46 j0	–	–
Glu^+^Cot/F	–	–	2.44 ± 0.51	–	1.00 ± 0.00

ChrA^+^Cot/F, Ins^+^Cot/F, and Glu^+^Cot/F – open type endocrine cells. SGls and DGls – superficial and deep glands, respectively. *p*-values ≤0.05 were statistically significant. The number of endocrine cells was defined as mean ± SD. **A** – Significant difference between DHC and DCHO; **B** – Significant difference between DHC and DCHI; **C** – Significant difference between DHC and DC; **D** – Significant difference between DHC and VF; **E** – Significant difference between DCHO and DCHI; **F** – Significant difference between DCHO and DC; **G** – Significant difference between DCHO and VF; **H** – Significant difference between DCHI and DC; **I** – Significant difference between DCHI and VF; **J** – Significant difference between DC and VF; **a** – Significant difference between SGls and DGls; **b** – Significant difference between ChrA^+^C/F and Ins^+^C/F; **c** – Significant difference between ChrA^+^C/F and Glu^+^C/F; **d** – Significant difference between Ins^+^C/F and Glu^+^C/F; **e** – Significant difference between ChrA^+^C/GL and Ins^+^C/GL; **f** – Significant difference between ChrA^+^C/GL and Glu^+^C/GL; **g** – Significant difference between Ins^+^C/GL and Glu^+^C/GL; **h** – Significant difference between ChrA^+^Cot/F and Ins^+^Cot/F; **i** – Significant difference between ChrA^+^Cot/F and Glu^+^Cot/F; **j** – Significant difference between Ins^+^Cot/F and Glu^+^Cot/F. **0, 1, 2, 3, 4** – absence of significance, *p* < 0.05, *p* < 0.01, *p* < 0.001, *p* < 0.0001, respectively.

The biliary epithelium lining the mucosal layer of the gallbladder and extrahepatic ducts was immunonegative. Only the cells of intramural glands of the studied organs were observed to be immunopositive (Figure 1). The density of ChrA^+^Cs per field was largest in DCHI, followed by DCHO, DHC, DC, and VB (Table 1).

### Immunoexpression of insulin and glucagon

The density of Ins^+^Cs and Glu^+^Cs was compared with that of ChrA^+^Cs to estimate the percentage of Ins^+^Cs and Glu^+^Cs. Immunohistochemical reactions for the detection of insulin and glucagon showed that the number of Ins^+^Cs and Glu^+^Cs was lower than the number of ChrA^+^Cs (Table 1). In the DHC, the density of Ins^+^Cs and Glu^+^Cs was almost equal, for example, Ins^+^Cs were 42% from all ChrA^+^Cs but Glu^+^Cs – 39%. In the DCHO, the number of Ins^+^Cs (48% from all ChrA^+^Cs) was significantly larger than Glu^+^Cs (28% from all ChrA^+^Cs). In the DCHI, the number of Ins^+^Cs (8% from all ChrA^+^Cs) and Glu^+^Cs (9% from all ChrA^+^Cs) was similar. In DC, the density of Ins^+^Cs was 89% from all ChrA^+^Cs but of Glu^+^Cs – 60%. In VB, the number of Ins^+^Cs, Glu^+^Cs, and ChrA^+^Cs was the same.

The largest number of Ins^+^Cs and Glu^+^Cs per field was detected in the DCHI, followed by the DCHO, DC, DHC, and VB (Table 1).

More ChrA^+^Cs per cross section of glands were observed in the superficial intramural glands than in the deep ones of the DCHO and DCHI while in the DHC, these cells in the deep glands were in higher abundance than in the superficial ones (Table 1). In the DC, the number of ChrA^+^Cs in the superficial and deep glands was the same.

Ins^+^Cs and Glu^+^Cs were found in the superficial glands only of the DHC, DCHO, and DC.

In the DCHI, Ins^+^Cs and Glu^+^Cs were observed in the superficial and deep glands. Their number was larger than in other ducts (Table 1).

### Open and closed type endocrine cells

In the VB, ChrA^+^Cs, and Ins^+^Cs were of closed type while Glu^+^Cs were of open type.

In the extrahepatic bile ducts, both open and closed types of ChrA^+^Cs were observed (Figure 1). In the DHC, the percentage of open type ChrA^+^Cs was 29% and those of closed type was 71%; in the DCHO, 33% of ChrA^+^Cs were open type and 67% were closed type; in the DCHI, 65% were open type and 35% were closed type; in the DC, 49% were open type and 51% were closed type.

Ins^+^Cs and Glu^+^Cs were represented by closed type only in the DHC, DCHO, DC, and VB.

In the DCHI, both open and closed types were present and their number was equal. The open type of Ins^+^Cs were 5.7% of the whole amount of open type ChrA^+^Cs, but open type Glu^+^Cs were 6.2% of the whole amount of open type ChrA^+^Cs.

In the connective tissue of the mucosal, muscular, and serosal layers of all studied organs, abundant chromogranin A-, glucagon-, and insulin-immunoreactive cells were detected but they were not described in this work because they are the object of our other as yet unpublished study (Figure 1).

## Discussion

In this study, porcine β- and α-cells were immunohistochemically identified for the first time in porcine DHC, DCH, DC, and VB. In our previous study (27), it was established that other endocrine cells are present in the porcine gallbladder and extrahepatic bile ducts such as ghrelin-, somatostatin-, serotonin-, and gastrin-positive endocrine cells but the data were not statistically analyzed. In the current study, we used the immunohistochemical detection of chromogranin A as a well-known marker for endocrine cells to identify the total number of endocrine cells in the DHC, DCHO, DC, and VB. As in our previous study (27), we revealed the localization of ChrA^+^Cs in the intramural glands of the studied organs, but unlike that study we statistically analyzed the distribution of ChrA^+^Cs per the superficial and deep glands. It is well known that ChrA regulates the secretory processes in an autocrine or paracrine manner. ChrA-positive cells were found to be chromaffin cells of the adrenal medulla, paraganglia, and entero-chromaffin-like cells and beta cells of the pancreas (28). Chromogranins including ChrA, chromogranin B, and secretogranin II are acidic proteins that have an important role in the formation of secretory granules in neuroendocrine cells (29, 30). Since chromogranins, including chromogranin A, are localized into neuroendocrine cells (31), they have been used as appropriate markers for this cell type in different organs. Helmant et al. (32) found that ChrA may be the prohormone of pancreastatin. We found out that the density of ChrA^+^Cs per microscopic field was the largest in porcine DCHI, followed by the DCHO, DHC, DC, and VB. The density of Ins^+^Cs and Glu^+^Cs per field of view in the same organs showed a similar manner of distribution.

Our finding regarding the ability of the biliary tract epithelium to produce extrapancreatic glucagon and insulin may contribute to improving the treatment of diabetes. To date, we have not been able to detect the presence of glucagon-secreting cells in the bile ducts and gall bladder of the domestic pig. Glucagon was primarily produced by the pancreatic α-cells using its precursor pro-glucagon (33, 34). There was no information about the glucagon-secreting cells in porcine bile ducts and gallbladder.

Pancreatic β-cells morphology and physiology have been well studied. There are data about the existence of extrapancreatic insulin-positive cells existing as single cells or groups of cells in and along the epithelium of the bile ducts, but not in the epithelium of the gallbladder. They are considered true β-cells because they produced insulin (35). There is evidence that some embryonic extrahepatic bile duct epithelial cells can be transformed into β-cells which are regulated by transcription factors such as Pdx1 (pancreatic and duodenal transcription factor 1 (36), HNF6 (37), and Hes1 (38)).

Regarding type 1 diabetic hyperglycemia, it is clear that several factors can cause it, such as the loss of β-cells and the postprandial increase of glucagon secretion from α-cells (39). Other authors have suggested that unsuitable glucagon secretion in type 1 diabetes depends on glucagon secretion directly from the gut (40).

Several mechanisms are known to prevent hypoglycemia, namely, reduced secretion of insulin from beta cells, reduced absorption of glucose in peripheral tissues, raised releasing of glucagon from α-cells, raised level of glucose, and a stimulated adrenal medulla. Defective α-cells and a reduced number of α-cells in type 1 diabetes alter glucagon responses (39).

The study by Dutton et al. (13) provided for the first time data on β-cells localization outside the pancreas in mammals, specifically mice. The authors found single β-cells among the cholangiocytes of the biliary mucosal epithelium, proving by measuring insulin mRNA that this type of endocrine cell arises precisely from the epithelium of the bile duct as early as the 17th embryonic day and their number increases up to 6 months after birth. Dutton et al. (13) also observed that clusters of cells appeared in the connective tissue layer of the extrahepatic bile ducts in the liver’s hilus, increasing in number after birth until 6 months of age. Regarding the presence of α-cells, these authors found single cells and a significantly lower number than β-cells. Dutton et al. (13) reported for the first time β cell formation from cholangiocytes.

Unlike Dutton et al. (13), we found that α- and β-cells were absent in the mucosal epithelium of all extrahepatic bile ducts. Such cells were observed in the biliary glands located in the propria of the mentioned organs, with the amount of β-cells approaching that of α-cells. Unlike Dutton et al. (13), we compared the amount of both types of cells in the different extrahepatic bile ducts and as a percentage of all endocrine cells positive for chromogranin A. For example, the highest number of Ins^+^Cs and Glu^+^Cs per field of view was found in the DCHI, followed by in the DCHO, DC, DHC, and VB. Ins^+^Cs and Glu^+^Cs were observed only in the superficial glands of the DHC, DCHO, and DC. In the DCHI, these cells were found in both superficial and deep glands and their number was highest, followed by DCHO, DHC, DC, and VB.

In the present study, elongated open-type endocrine cells and oval closed-type endocrine cells were observed, which correlated with the findings of a number of authors regarding the shape of intestinal endocrine cells (35–37). It is known that intestinal endocrine cells of open type contact the luminal content, where they react to stimuli from their apical membrane receptors (33, 34), whereas the closed type of endocrine cells do not reach the lumen and have a paracrine manner of action on the surrounding target cells (35). In the current study, it was revealed that the three types of endocrine cells (ChrA^+^Cs, Ins^+^Cs, and Glu^+^Cs) in the VB were of closed type. However, in the extrahepatic bile ducts, both open and closed type of ChrA^+^Cs were observed. Ins^+^Cs and Glu^+^Cs were represented by closed type only in the DHC, DCHO, DC, and VB. In the DCHI, both open and closed Ins^+^Cs and Glu^+^Cs types were present and their number was equal. Therefore, the secretory products of open cell type acted locally or on distant target cells through the bloodstream. The cells of closed type were localized between other epithelial cells which means that, like intestinal closed cell types, they do not interact directly with luminal substances (35–37).

The presence of glucagon- and insulin-positive endocrine cells in porcine bile ducts and gallbladder can be explained by studies by other authors (38, 39) that have revealed the key role of transcription factor Sox9, the activation of which leads to the transition of pancreatic progenitor cells to mature endocrine cells. For example, in mice and humans, endocrine cell differentiation has been shown to be primarily regulated by Sox9 relaying on the PI3K/Akt signaling pathway (39).

According to Banga et al. (38), Sox9^+^ liver cells are represented by small bile ducts, hepatoblast-like progenitors in the periportal area, or peribiliary glands within larger bile ducts. Thus, Banga et al. (38) provided evidence of the *in vivo* reprogramming of bile duct cells to a β-cell-like phenotype that can relieve diabetes in adult individuals.

Lund et al. (40) revealed that extrapancreatic glucagon and observed postprandial hyperglucagonemia in total pancreatectomized patients may have clinical and scientific applications. These authors found that the glucagon secreted from extrapancreatic tissue in humans changed the concept of glucagon as a pancreas-specific hormone and thus opened the way to a new explanation of postprandial hyperglucagonemia, as it may be a gut-dependent phenomenon. Lund et al. (40) suggested that gut-derived glucagon might play a previously unknown role in secondary diabetes, following pancreatectomy, and possibly, although speculatively, also in the pathophysiology of other conditions following dietary hyperglucagonemia, including type 2 diabetes.

We supposed that the presence of glucagon-producing cells in porcine extrahepatic bile ducts and gallbladder was probably related to the ability of this hormone to perform the same function in these organs like gut. Based on the results of our research, we hypothesize that there is a functional connection between the extrahepatic bile ducts and the islets of Langerhans which can be defined as a biliary-islet axis, similar to the functional connection between the intestine and the islets of Langerhans named by Fehmann et al. (41) the entero-islet axis. The therapy of type 1 diabetes by means of pancreatic islet transplantation has been investigated for many years and applied with increasing success, but the number of transplants was limited due to the limited supply of donors (19). That is why alternative ways to synthesize β-cells has been sought (20). Several studies have shown that hepatocytes (26) and pancreatic acinar cells (42) can transform into β-cells. These findings define the biliary endocrine cells as a component of the gastroenteropancreatic endocrine system and as another source of glucagon and insulin, which could be used in the development of new approaches in the treatment of diabetes mellitus. Endocrine cells in pancreatic excretory ducts release insulin, glucagon, somatostatin, and pancreatic polypeptide which might regulate the function of rat pancreatic acinar cells (43) as well as influence bile production (44). Diabetes mellitus influences duct endocrine cell function and alters cholesterol metabolism in the direction of stone formation in bile and pancreatic ducts (45, 46). Glucagon positive cells in gut and extrahepatic bile ducts participate in functional connection between these organs and pancreatic islands.

## Conclusion

The original distribution of our study was identification of porcine β- and α-cells in porcine extrahepatic bile ducts and gallbladder. The largest number of glucagon- and insulin-positive cells was detected in the intramural part of the DCH, followed by its extramural part, the DC, DHC, and VB. These findings defined biliary endocrine cells as components of the gastroenteropancreatic endocrine system and as another source of glucagon and insulin, which can be used in the development of new approaches in the treatment of diabetes mellitus.

## Data availability statement

The raw data supporting the conclusions of this article will be made available by the authors, without undue reservation.

## Ethics statement

The animal study was approved by Ethics committee of the Bulgarian Food Safety Agency. The study was conducted in accordance with the local legislation and institutional requirements.

## Author contributions

IS: Conceptualization. IS, MG: Methodology. IS: Software, Formal analysis. IS: Investigation. IS: Data curation. IS: Writing - original draft preparation. IS, SS, and MG: Writing - review and editing. IS and SS: Visualization. IS: Supervision. IS: Project administration. All authors have read and agreed with the published version of the manuscript.
